# Action channel switching costs in the comprehension of concrete and abstract mandarin verbs

**DOI:** 10.3389/fpsyg.2026.1796806

**Published:** 2026-04-28

**Authors:** Zhiwei Cai, Zhongyuan Qu, Ning Fan

**Affiliations:** 1 College of Teacher Education, Baoding University, Baoding, Hebei, China; 2 College of Education, Hebei University, Baoding, Hebei, China

**Keywords:** abstractness, action channel switching cost, concreteness, perceptual symbol systems theory, words as social tools theory

## Abstract

The grounding of language in perceptual–motor systems remain a central issue in embodied cognition research, particularly regarding whether abstract concepts engage sensorimotor representations in a manner similar to concrete concepts. The present study employed a modality-switch paradigm to investigate, across three experiments, whether readers engage in embodied mental simulation when comprehending concrete and abstract verbs related to eye-, mouth-, and hand-action channels. The results showed that action channel switching costs emerged for verb phrases consisting of concrete verbs paired with concrete nouns, concrete verbs paired with abstract nouns, and abstract verbs paired with abstract nouns, although the effects were manifested in different action channels. Taken together, the findings indicate that the comprehension of both concrete and abstract verbs relies on perceptual–motor simulation in the corresponding action channels. The results are consistent with the Perceptual Symbol Systems theory, which accounts for the channel-specific switch costs.

## 1 Introduction

Humans interact with the external world through different perceptual and motor channels and use language to describe the properties of objects perceived through these channels. For example, visual features of objects are accessed via the visual channel and described using words such as red, whereas sounds produced by objects are accessed via the auditory channel and described using words such as noisy. Research has shown that readers process linguistic information in a modality-specific manner, and that switching between modalities incurs a processing cost, known as the modality-switch effect. This effect is typically manifested as increased error rates or longer reaction times (Pecher et al., 2003; Scerrati et al., 2015; Vermeulen et al., 2007). Specifically, when readers process two consecutively presented phrases or sentences, responses to the second phrase or sentence are slower if it involves a different perceptual modality from the first (e.g., visual–auditory).

Studies on verbs and action sentences have revealed a similar phenomenon of action-channel specificity. When the semantics of an action sentence (e.g., push) are compatible with the required motor response (e.g., extending the arm), a compatibility effect occurs, facilitating responses (Cai et al., 2025; Fan et al., 2026; Ghandhari et al., 2020; Glenberg and Kaschak, 2002). In contrast, when the action described in the sentence (e.g., grasp) is unrelated to the motor response (e.g., clap) but engages the same action channel (e.g., the hand), interference effects arise due to channel occupation, leading to slower reaction times (Bergen et al., 2003; Schaller et al., 2017).

To account for these phenomena, the Perceptual Symbol Systems (PSS) theory within the embodied cognition framework proposes that language comprehension is grounded in perceptual–motor simulation, reactivating perceptual and motor experiences across multiple modalities (Barsalou, 1999, 2020). Neuroimaging studies have shown that understanding verbs, imagining actions, and performing actual actions activate overlapping motor networks in the brain (Papeo et al., 2009), and that the degree of effort implied by an action in a sentence is related to the level of activation in the motor system (Moody and Gennari, 2010). Subsequent studies have further demonstrated that, analogous to modality-switch costs between perceptual channels, switching costs also exist between motor channels such as the hands and feet (Awazu, 2011). Together, these findings provide converging evidence that language comprehension is modality- and action-channel specific.

Unlike concrete actions (e.g., 推动桌子/push a table), abstract actions (e.g., 推动发展/promote development) lack clear perceptual–motor referents. A key question therefore remains: Does the comprehension of abstract actions still activate motor experiences similar to those involved in concrete actions?

The Words as Social Tools (WAT) theory proposes that concrete concepts rely more heavily on the external environment and are constructed from perceptual–motor experiences with the physical world, whereas abstract concepts are acquired primarily through social interaction, especially via mouth-related motor activity involved in communication with others (Wauters et al., 2003). Although both concrete and abstract concepts are grounded in perceptual–motor experience, abstract concepts are assumed to engage the mouth motor channel to a greater extent (Borghi et al., 2011, 2019; Borghi and Binkofski, 2014; Mazzuca et al., 2025). Empirical studies have shown that, compared with concrete concepts, the mouth exhibits a response advantage for abstract—but not concrete—concepts (Granito et al., 2015), influencing the processing of abstract verbs and abstract concepts but not concrete concepts (Borghi et al., 2019; Schaller et al., 2017). Accordingly, this theory predicts that the comprehension of abstract actions relies primarily on the mouth motor channel and that no switching costs should occur between different action channels.

In contrast, the PSS theory argues that the comprehension of abstract and concrete concepts follows similar mechanisms, both relying on perceptual–motor simulation, with abstract concepts engaging sensorimotor simulation across a broader range of modalities (D’Angiulli et al., 2015). Specifically, perceptual–motor simulation refers to the partial reactivation of sensory and motor experiences associated with a concept, including visual (e.g., shape or spatial orientation), auditory (e.g., sound-related features), and motoric components (e.g., effector-specific actions such as hand or mouth movements). During language comprehension, these modality-specific representations can be automatically and rapidly activated.

Consistent with this view, previous studies have shown that both concrete and abstract language can elicit action-related compatibility effects. Glenberg and Kaschak (2002) reported the Action–Sentence Compatibility Effect (ACE), a phenomenon in which participants respond faster when the direction of a required motor response is congruent with the action direction implied by a sentence than when it is incongruent. For example, when judging the sensibility of sentences implying object transfer, responses are facilitated if the response movement matches the direction encoded in the sentence. Importantly, similar effects have been observed not only for sentences describing concrete object transfer (e.g., “open the drawer”), but also for sentences involving more abstract transfer, suggesting that even abstract linguistic constructions can recruit motor simulations. In addition, studies using phrases composed of concrete verbs combined with abstract nouns have found that readers still engage in motor simulation during comprehension (Pambuccian, 2021; Reilly et al., 2019). These findings suggest that the understanding of abstract verbs may still depend on specific action channels and that switching costs between action channels may therefore persist.

In summary, the PSS theory and the WAT theory offer different accounts of the embodied representation of abstract verbs and generate contrasting predictions regarding action-channel switching costs for abstract actions. Importantly, however, the difference between the two theories may not be strictly dichotomous. Even if abstract actions rely preferentially on the mouth motor system, such reliance may not be entirely exclusive. The presence of switching costs for abstract actions would not automatically falsify the WAT account, but rather call for a more nuanced interpretation concerning the degree and distribution of motor channel involvement. Most existing studies have focused on the hand and foot motor channels, leaving it unclear whether switching costs also occur in other action channels such as the mouth and eyes. Therefore, the present study employs verb phrases involving three action channels—eye, hand, and mouth—and adopts a channel-switching paradigm to examine action-channel switching costs in the comprehension of concrete and abstract verbs, thereby testing the competing predictions of the two theories.

Furthermore, following the general approach of Schaller et al. (2017), who manipulated verb abstractness by embedding verbs in sentential contexts, the present study adopts verb–object phrases to operationalize the semantic abstractness of verbs. Compared to full sentences, verb phrases reduce potential confounding factors such as subject identity, grammatical person, and syntactic complexity, thereby allowing for a more controlled examination of verb semantics. For instance, previous research has shown that reading action sentences with second- or third-person pronouns can elicit different perspectives in motor simulation (Cai et al., 2025), and that sentence-level features such as syntactic structure (e.g., double-object constructions; Glenberg and Kaschak, 2002) and tense (Bergen and Wheeler, 2010) can also influence how readers simulate actions. At the same time, the object nouns following the verbs serve as a critical source of contextual information, enabling systematic manipulation of the degree of semantic abstractness while minimizing irrelevant linguistic variation. Therefore, the present study employs verb–object phrases, rather than isolated verbs or full sentences, as experimental materials.

The study comprises three experiments. Experiment 1 examines action-channel switching costs when concrete verbs are combined with concrete nouns. Experiment 2 examines action-channel switching costs when concrete verbs are combined with abstract nouns. Experiment 3 examines action-channel switching costs when abstract verbs are combined with abstract nouns. Under a strong interpretation of the WAT framework, switching costs for abstract verbs should be minimal or absent, given the assumed primary reliance on the mouth motor channel. Previous empirical findings have tended to suggest an all-or-none pattern; however, these studies have examined only a limited range of motor channels and have not systematically explored the full spectrum of concrete–abstract verb combinations. If abstract concepts engage multiple motor channels to some extent, switching costs may be attenuated rather than completely eliminated. In contrast, the PSS theory predicts that both concrete and abstract verbs rely on modality- and channel-specific sensorimotor simulation; therefore, switching costs between the eye, hand, and mouth channels should be observed during the comprehension of abstract verbs as well.

## 2 Experiment 1: Action-channel switching costs in concrete verb–noun phrases

### 2.1 Method

#### 2.1.1 Participants

Participant sample size was determined based on an *a priori* power analysis conducted using G*Power 3.1, assuming a medium effect size (Effect size *f* = 0.25), *α* = 0.05, and desired power (1 − *β*) = 0.95, which indicated a minimum required sample size of 28 participants. Thirty-eight undergraduate students (14 males, 24 females) were recruited from a university. The mean age was 21.62 ± 2.14 years. All participants had normal or corrected-to-normal vision and had not taken part in similar experiments. They received a small monetary compensation after the experiment. The same criteria applied to the subsequent experiments. The study was approved by the Ethics Committee of Affiliated Hospital of Hebei University (protocol code: HDFY-LL-2021-178). All participants provided written informed consent prior to the experiment in accordance with the Declaration of Helsinki. The same ethical procedure was applied to the subsequent experiments.

#### 2.1.2 Design

The experiment adopted a 3 (Channel Type: eye, hand, mouth) × 2 (Switching Type: no switch, switch) within-subjects design. Reaction times in a phrase plausibility judgment task served as dependent variables.

#### 2.1.3 Materials

A total of 378 two-character Chinese concrete and abstract verbs related to the eye, hand, and mouth were selected from the Modern Chinese Verb Classification Dictionary and the Modern Chinese Classified Dictionary. Thirty-five undergraduate students who did not participate in the experiment rated the concreteness/abstractness of these verbs on a 9-point scale (1 = very abstract, 9 = very concrete). After excluding three participants who did not complete the questionnaire seriously, the data were analyzed. Verbs with a mean rating below 4 were classified as abstract verbs, and those with a mean rating above 6 were classified as concrete verbs. This procedure yielded 29 eye-related concrete verbs, 31 mouth-related concrete verbs, and 99 hand-related concrete verbs, as well as 21 eye-related abstract verbs, 20 mouth-related abstract verbs, and 22 hand-related abstract verbs.

These verbs were combined with concrete or abstract nouns to create 350 semantically plausible verb phrases for further selection. In addition, 250 implausible phrases were constructed. Another group of 30 undergraduate students rated the plausibility, familiarity, and emotional valence of all phrases using a 5-point scale. Phrases with a standard deviation greater than 1.5 were excluded. Phrases with a mean plausibility rating above 4 were defined as plausible phrases, whereas those with a mean rating below 2 were defined as implausible phrases. Subsequently, 40 undergraduate students rated the concreteness/abstractness of the plausible phrases on a 9-point scale and assessed channel appropriateness using a forced-choice task. Phrases with a standard deviation greater than 2.5 on concreteness/abstractness or with unclear channel assignment (i.e., fewer than 90% of participants selecting the same channel) were excluded. The remaining phrases constituted the candidate materials for the experiment.

From the candidate phrases consisting of concrete verbs paired with concrete nouns, phrases were matched across the three channels in terms of concreteness/abstractness, total number of strokes (i.e., the total number of brush/pen strokes required to write the Chinese characters in the phrase, which reflects visual-orthographic complexity), plausibility, emotional valence, and familiarity (*Fs* ≤ 1.90, *ps* ≥ 0.31). In addition, lexical frequency was controlled at the word level. Based on the Chinese Lexical Database (Sun et al., 2018), analyses of variance showed that the frequencies of verbs, *F* (2, 45) = 1.256, *p* = 0.295, and nouns, *F* (2, 45) = 1.059, *p* = 0.355, did not differ significantly across the three types of phrases. Likewise, concreteness was balanced across conditions, with no significant differences observed for verbs, *F* (2, 45) = 1.609, *p* = 0.211, or nouns, *F* (2, 45) = 0.976, *p* = 0.384. Sixteen phrases were selected for each channel, such as 观察蚂蚁/observing ants (eye channel), 擦拭桌子/wiping the table (hand channel), and 咀嚼香蕉/chewing a banana (mouth channel). Descriptive statistics for each dimension are presented in Table 1. Sixteen plausible filler phrases with unclear channel assignment (e.g., 倾佩勇气/admire courage) were included but not analyzed. In addition, 100 implausible phrases (e.g., 相信长裤/believe trousers) were included as catch trials to balance participants’ response tendencies.

**Table 1:** Matching results for phrases across different channels in Experiment 1 (*M* ± *SD*).

Channel type	Concreteness	Number of strokes	Plausibility	Emotional valence	Familiarity
Eye-related	8.15 ± 0.29	33.19 ± 5.36	4.50 ± 0.23	3.49 ± 0.33	4.14 ± 0.40
Hand-related	8.34 ± 0.15	33.13 ± 5.40	4.66 ± 0.26	3.06 ± 0.92	4.32 ± 0.37
Mouth-related	8.24 ± 0.35	33.94 ± 8.94	4.57 ± 0.32	3.30 ± 0.65	4.22 ± 0.52

#### 2.1.4 Procedure

The experiment was conducted in a quiet laboratory using a desktop computer with a 20-inch LCD monitor. Experimental stimuli were presented with E-Prime 2.0 in white font on a black background. Before the formal experiment, participants completed sufficient practice trials to become familiar with the procedure.

Each trial began with a fixation cross (“+”) presented at the center of the screen for 300 ms, followed by a blank screen for 300 ms. A phrase (e.g., 品尝甜点/taste dessert) then appeared at the center of the screen. Participants were instructed to judge the plausibility of the phrase by pressing the “Q” or “P” key using the index fingers of both hands. The phrase disappeared either when a response was made or after 2000 ms, followed by a 1,000-ms blank screen. The next trial then began.

If the subsequent trial was “亲吻脸颊”/kiss the cheek, it belonged to the same oral action channel as the previous trial (品尝甜点/taste dessert) and was classified as a no-switch condition. If the subsequent trial was “遥望夜空”/gaze at the night sky, it involved a different action channel (mouth–visual) and was classified as a switch condition.

The experiment consisted of two blocks, each containing 200 trials, with plausible and implausible phrases presented in equal proportions. A pseudo-random sequence was used to arrange the order of action channels, ensuring an equal number of switch and no-switch trials for each channel. Within each channel, phrase order was randomly selected by the computer. The assignment of phrases was counterbalanced across the two blocks so that each phrase served once as a no-switch target in one block and once as a switch target in the other block. Three versions of pseudo-random sequences were created and counterbalanced across participants. Response key mappings (left/right hand) were also counterbalanced across participants.

Across the two blocks, a total of 96 plausible trials were included in the data analysis, with 32 trials for each action channel (visual, manual, and mouth) and equal numbers of switch and no-switch conditions. A switch trial refers to a trial in which the current phrase involves a different dominant action channel than that of the preceding trial, whereas a no-switch trial involves repetition of the same channel across consecutive trials. The entire experiment took approximately 15 min to complete.

### 2.2 Results

First, data from two participants with accuracy rates below 80% were excluded. Trials corresponding to filler sentences were then removed from the analysis. Next, trials with incorrect responses were excluded. Reaction times shorter than 400 ms or longer than 1,500 ms, as well as extreme values beyond ±3 *SD*, were then removed.

Given the repeated-measures structure of the design, reaction times was analyzed using linear mixed-effects models (LMMs). The fixed effects included Channel Type (three levels: eye, hand, and mouth), Switch Type (two levels: switch vs. no-switch), and their interaction. Participants and stimuli were included as random effects with random intercepts. This specification allowed us to account for potential variability associated with both individual participants and item-specific characteristics. When significant interactions were observed, planned comparisons and simple effects analyses were conducted using estimated marginal means.

To examine potential channel-specific switch costs, separate Bayesian models were constructed for each channel using the *BayesFactor* package in R (Morey and Rouder, 2015). For each channel, a full model including the Switch effect was compared against a corresponding null model without the Switch term. This procedure allowed us to directly test whether a reliable switch cost was present within each specific channel. Competing models differing in the presence or absence of specific fixed effects (e.g., the Channel × Switch interaction) were compared using Bayes factors based on JZS priors. Bayes factors were interpreted following conventional benchmarks, with values greater than 3 indicating moderate evidence for the alternative model and values below 1/3 indicating moderate evidence for the null model (Wagenmakers et al., 2018).

The random-effects results indicated that the variance of participant intercepts was 7,728 (*SD* = 88), the variance of item intercepts was 3,102 (*SD* = 56), and the residual variance was 33,352 (*SD* = 183), suggesting that both individual differences and item differences contributed to the overall variability in reaction times, although the residuals remained the largest source of variance.

For the fixed-effects, there was no significant main effect of Channel Type (*ps* > 0.63), whereas Switching Type showed a significant effect, *β* = 0.112, *t* = 2.021, *p* = 0.043, *CI* = [0.003, 0.221].

The interaction between Channel Type and Switching Type was not significant for the mouth condition, *β* = −0.093, *t* = 0.234, *p* = 0.815, *CI* = [−0.245, 0.059], nor for the hand condition, *β* = 0.018, *t* = −1.202, *p* = 0.229, *CI* = [−0.133, 0.169]. Post-hoc comparisons revealed that in the Eye condition, reaction times were significantly shorter for no-switch trials than switch trials (*t* = −2.021, *p* = 0.043), and in the Hand condition, no-switch trials were also faster than switch trials (*t* = −2.437, *p* = 0.014). No significant difference was observed in the Mouth condition (*t* = −0.354, *p* = 0.723).

Bayesian model comparisons provided converging evidence regarding the absence of channel-specific switch effects. For the mouth channel, the Bayes factor (BF₁₀ = 0.112, ±1.23%) indicated moderate-to-strong evidence in favor of the null model, suggesting that including a switch effect did not improve model fit. For the eye channel, BF₁₀ = 0.529 (±1.69%) yielded only anecdotal evidence against the presence of a switch cost, providing no substantial support for either model. Similarly, for the hand channel, BF₁₀ = 0.753 (±1.24%) indicated weak evidence favoring the null model. Taken together, these results suggest that the data provide moderate evidence against a mouth-specific switch effect and remain inconclusive for the eye and hand channels (Table 2).

**Table 2:** Mean reaction times and error rates for different action channels in Experiment 1 (*M* ± *SD*).

Channel type	RT (*ms*)		Error rate (*%*)	
	No-switch	Switch	No-switch	Switch
Eye-related	750 ± 89	775 ± 96	4.81 ± 6.49	6.86 ± 6.66
Hand-related	748 ± 95	775 ± 88	5.03 ± 5.35	5.73 ± 5.66
Mouth-related	760 ± 118	764 ± 111	6.08 ± 7.39	7.47 ± 8.56

## 3 Experiment 2: Action-channel switching costs in concrete verb–abstract noun phrases

### 3.1 Method

#### 3.1.1 Participants

Thirty-seven undergraduate students were recruited from a university, none of whom had participated in similar experiments. The sample included 13 males and 24 females, with a mean age of 22.33 ± 1.91 years.

#### 3.1.2 Design

The experiment adopted a 3 (Channel Type: eye, hand, mouth) × 2 (Switching Type: no switch, switch) within-subjects design. Reaction times in a phrase plausibility judgment task served as dependent variables.

#### 3.1.3 Materials

From the phrases combining concrete verbs with abstract nouns, items were matched on concreteness/abstractness, total number of strokes, plausibility, emotional valence, and familiarity (*Fs* ≤ 1.73, *ps* ≥ 0.20). Analyses of variance showed that the frequencies of verbs, *F*(2, 21) = 0.271, *p* = 0.765, and nouns, *F*(2, 21) = 0.405 *p* = 0.672, did not differ significantly across the three types of phrases. Similarly, concreteness was balanced across conditions, with no significant differences observed for verbs, *F*(2, 21) = 1.971, *p* = 0.164, or nouns, *F*(2, 21) = 2.538, *p* = 0.103. This resulted in 8 phrases for each of the three channels. Descriptive statistics are presented in Table 3. In addition, 8 plausible filler phrases were included but not entered into the formal analyses, and 50 implausible phrases were used as error trials to balance participants’ response keys.

**Table 3:** Matching results for phrases across different channels in Experiment 2 (*M* ± *SD*).

Channel type	Concreteness	Number of strokes	Plausibility	Emotional valence	Familiarity
Eye-related	2.94 ± 0.58	34.25 ± 5.33	4.35 ± 0.13	3.77 ± 0.72	4.04 ± 0.84
Hand-related	2.75 ± 0.32	37.13 ± 6.68	4.36 ± 0.17	3.96 ± 0.74	4.26 ± 0.38
Mouth-related	3.25 ± 0.65	35.50 ± 8.07	4.29 ± 0.12	3.24 ± 1.52	3.84 ± 0.32

#### 3.1.4 Procedure

The procedure was identical to that of Experiment 1. Each block contained 100 trials, with an equal number of plausible and implausible phrases. Across the two blocks, a total of 48 plausible trials were included in the data analysis, with 16 trials for each of the three channels and an equal number of switch and no-switch conditions. The entire experiment took approximately 8 min to complete.

### 3.2 Results

Data preprocessing and analysis followed the same procedures as in Experiment 1. One participant with an accuracy rate below 80% was excluded. Descriptive statistics for each condition are presented in Table 4.

**Table 4:** Mean reaction times and error rates across different action channels in Experiment 2(*M ± SD*).

Channel type	RT(*ms*)		Error rate (*%*)	
	No-switch	Switch	No-switch	Switch
Eye-related	778 ± 113	826 ± 128	3.57 ± 7.14	3.57 ± 6.27
Hand-related	829 ± 127	820 ± 123	4.36 ± 8.92	7.93 ± 10.49
Mouth-related	787 ± 114	830 ± 154	9.52 ± 10.24	6.74 ± 9.34

The random-effects results indicated that the variance of participant intercepts was 8,153 (*SD* = 90), the variance of item intercepts was 597 (*SD* = 24), and the residual variance was 58,581 (*SD* = 242), suggesting that both individual differences and item differences contributed to the overall variability in reaction times, although the residuals remained the largest source of variance.

For the fixed-effects, there was significant main effect of Channel Type for the hand condition, with longer reaction times than the eye condition (*β* = 0.200, *t* = 2.024, *p* = 0.049, *CI* = [0.006, 0.393]), while the mouth condition did not differ from the eye condition (*β* = 0.036, *t* = 0.360, *p* = 0.720, *CI* = [−0.160, 0.232]). Switch Type also showed a significant main effect, with longer reaction times on switch trials compared to no-switch trials (*β* = 0.187, *t* = 2.2026, *p* = 0.028, *CI* = [0.021, 0.353]).

The interaction between Channel Type and Switching Type was not significant for either the mouth condition (*β* = −0.027, *t* = −0.226, *p* = 0.821, *CI* = [−0.265, 0.211]) or the hand condition (*β* = −0.213, *t* = −1.770, *p* = 0.077, *CI* = [−0.450, 0.023]). Post-hoc comparisons revealed that in the eye condition, no-switch trials were significantly faster than switch trials (*t* = −2.206, *p* = 0.028), whereas in the hand condition (*t* = 0.310, *p* = 0.757) and mouth condition (*t* = −1.836, *p* = 0.067), the differences were not significant.

Bayesian model comparisons provided converging evidence regarding channel-specific switch effects. For the mouth channel, the Bayes factor (BF₁₀ = 0.455, ±1.69%) indicated only anecdotal evidence in favor of the null model, suggesting that the data were insensitive and did not clearly distinguish between the presence and absence of a switch effect. For the eye channel, BF₁₀ = 0.921 (±1.00%) was close to 1, likewise indicating negligible evidence for either model and thus no reliable support for a switch cost. In contrast, for the hand channel, BF₁₀ = 0.140 (±1.73%) provided moderate evidence in favor of the null model, suggesting that including a switch term did not improve model fit. Taken together, the Bayesian results offer no compelling evidence for channel-specific switch costs, with moderate support for the absence of such an effect in the hand channel and largely inconclusive evidence for the mouth and eye channels.

## 4 Experiment 3: Action-channel switching costs in abstract verb–noun phrases

### 4.1 Method

#### 4.1.1 Participants

Thirty-eight university students (13 males, 25 females, mean age = 21.95 ± 2.43 years) who had not participated in similar experiments were recruited.

#### 4.1.2 Design

The experiment adopted a 3 (Channel Type: eye, hand, mouth) × 2 (Switching Type: no switch, switch) within-subjects design. Reaction times in a phrase plausibility judgment task served as dependent variables.

#### 4.1.3 Materials

From the set of phrases combining abstract verbs with abstract nouns, phrases were matched on concreteness/abstractness, total stroke count, plausibility, emotional valence, and familiarity (*Fs* ≤ 2.209, *ps* ≥ 0.100), resulting in 12 phrases for each of the three channels. In addition, analyses of variance indicated that the frequencies of both verbs, *F*(2, 33) = 1.022, *p* = 0.371, and nouns, *F*(2, 33) = 1.469, *p* = 0.245, did not differ significantly across the three phrase types. Likewise, concreteness was comparable across conditions, with no significant differences observed for verbs, *F*(2, 33) = 0.076, *p* = 0.927, or nouns, *F*(2, 33) = 2.087, *p* = 0.140. Descriptive statistics are shown in Table 5. Additionally, 16 plausible filler phrases were included but not analyzed, and 100 implausible phrases were included to balance participants’ key responses for error trials.

**Table 5:** Matching results for phrases across different channels (*M* ± *SD*).

Channel type	Concreteness	number of strokes	Plausibility	Emotional valence	Plausibility
Eye-related	2.90 ± 0.52	31.16 ± 5.49	4.71 ± 0.22	3.03 ± 1.25	4.33 ± 0.37
Hand-related	2.84 ± 0.52	32.75 ± 5.36	4.67 ± 0.25	3.46 ± 0.76	4.40 ± 0.43
Mouth-related	3.19 ± 0.60	34.83 ± 5.73	4.48 ± 0.37	3.13 ± 1.44	4.24 ± 0.42

#### 4.1.4 Procedure

The experimental procedure was the same as in Experiment 1. Each block contained 200 trials, with half plausible and half implausible phrases. Across the two blocks, 72 plausible trials were used for data analysis, with 24 phrases for each of the three channels, and an equal number of trials in the switch and no-switch conditions. The entire experiment took approximately 15 min to complete.

### 4.2 Results

Data preprocessing and analysis followed the same procedures as in Experiment 1. Two participants with excessively high error rates were excluded from the analysis. Descriptive statistics for each condition are presented in Table 6.

**Table 6:** Mean reaction times and error rates for different action channels in Experiment 3 (*M ± SD*).

Channel type	RT(*ms*)		Error rate (*%*)	
	No-switch	Switch	No-switch	Switch
Eye-related	737 ± 102	738 ± 104	2.53 ± 4.67	3.28 ± 6.20
Hand-related	730 ± 88	761 ± 108	4.86 ± 6.42	7.18 ± 8.72
Mouth-related	712 ± 88	759 ± 117	6.79 ± 8.52	4.63 ± 6.16

The random-effects results indicated that the variance of participant intercepts was 7,029 (*SD* = 84), the variance of item intercepts was 5,114 (*SD* = 72), and the residual variance was 32,451 (*SD* = 180), suggesting that both individual differences and item differences contributed to the overall variability in reaction times, although the residuals remained the largest source of variance.

For the fixed-effects, there was not significant main effect of channel type. Reaction times in the hand condition did not differ from the eye condition (*β* = −0.014, *t* = −0.090, *p* = 0.929, *CI* = [−0.318, 0.234]), nor did the mouth condition differ from the eye condition (*β* = −0.080, *t* = −0.479, *p* = 0.635, *CI* = [−0.408, 0.248]). Switch Type also did not produce a significant main effect, with slightly longer reaction times on switch trials relative to no-switch trials (*β* = 0.035, *t* = 0.556, *p* = 0.578, *CI* = [−0.088, 0.157]).

The Channel × Switch interaction was significant for the mouth condition (*β* = 0.111, *t* = 2.028, *p* = 0.043, *CI* = [−0.060, 0.282]), but not for the hand condition (*β* = 0.190, *t* = 1.273, *p* = 0.203, *CI* = [0.006, 0.375]). Post-hoc comparisons indicated that in the eye condition, no-switch trials were not significantly faster than switch trials (*t* = −0.556, *p* = 0.578), whereas significant switch costs were observed in both the hand (*t* = −2.393, *p* = 0.017) and mouth (*t* = −3.212, *p* = 0.001) conditions.

Bayesian model comparisons yielded differentiated evidence across channels. For the mouth channel, the Bayes factor (BF₁₀ = 3.369, ±1.36%) indicated moderate evidence in favor of the alternative model, suggesting that including a switch effect improved model fit and providing support for a mouth-specific switch cost. In contrast, for the eye channel, BF₁₀ = 0.117 (±1.45%) provided moderate-to-strong evidence in favor of the null model, indicating that a switch effect was unlikely in this channel. For the hand channel, BF₁₀ = 0.873 (±2.10%) was close to 1, reflecting only anecdotal evidence and thus failing to clearly distinguish between the presence and absence of a switch cost. Taken together, these results suggest evidence for a switch effect in the mouth channel, moderate evidence against such an effect in the eye channel, and inconclusive evidence in the hand channel.

## 5 General discussion

This study employed verb phrases corresponding to specific effector channels and used a modality-switch paradigm (Scerrati et al., 2015; Vermeulen et al., 2007) to examine the embodied cognitive processes in understanding concrete and abstract verbs, thereby testing the Perceptual Symbol Systems theory and the Words as Social Tools theory. Across the three experiments, the overall pattern of results revealed differentiated channel-specific effects rather than a uniform switching pattern. In Experiment 1 (concrete verbs + concrete nouns), switching costs emerged behaviorally in the eye and hand channels, although Bayesian analyses did not provide strong evidence for robust channel-specific effects. In Experiment 2 (concrete verbs + abstract nouns), a general switching cost was observed, mainly driven by the eye channel, with Bayesian results offering limited or inconclusive support for channel-specific effects. In Experiment 3 (abstract verbs + abstract nouns), a significant Channel × Switch interaction was found for the mouth channel, and Bayesian analyses provided moderate evidence for a mouth-specific switching cost, alongside evidence against such an effect in the eye channel.

Taken together, these findings suggest that switching costs were neither universally present nor entirely absent across conditions. Instead, the magnitude of switching effects varied as a function of both verb type and effector channel, indicating that motor system involvement during verb comprehension is not an all-or-none phenomenon for either concrete or abstract actions.

### 5.1 Theoretical implications for embodied verb processing

According to PSS theory, language comprehension involves reactivating multiple perceptual and motor experiences. Previous studies using concrete linguistic materials have observed modality-switch costs across visual–auditory channels (Pecher et al., 2003, 2004; Posner and DiGirolamo, 2000), perceptual-emotional channels (Vermeulen et al., 2007), and hand-foot action channels (Awazu, 2011). In the present study, concrete verb phrases showed modality-switch costs in the eye and hand channels (Experiment 1), consistent with Scerrati et al. (2015), supporting PSS theory. Moreover, when concrete verbs were paired with abstract nouns (Experiment 2), a general switching cost emerged, mainly in the eye channel, further indicating that perceptual-motor simulations contribute to comprehension across modalities. Crucially, abstract verb phrases paired with abstract nouns (Experiment 3) exhibited a significant Channel × Switch interaction for the mouth channel, with Bayesian analyses providing moderate evidence for a mouth-specific switch cost and evidence against a switch cost in the eye channel. These findings indicate that even abstract verb comprehension engages specific perceptual-motor channels, consistent with PSS theory, and suggest that motor simulation is not limited to concrete actions but operates in a channel-specific manner across both concrete and abstract verbs.

The WAT theory posits that abstract concepts are primarily embodied in linguistic experience and social learning. Unlike concrete concepts, which are directly embodied in the individual’s sensorimotor system through perceptual and motor experiences, abstract concepts rely more heavily on language input and are closely associated with the motor system involved in speech production, particularly the mouth (Wauters et al., 2003). While concrete concepts are learned with clear actions or referents, abstract concepts are acquired through interaction and communication. Since spoken language is the typical medium for expressing words, abstract concepts preferentially activate the mouth-related motor system (Borghi et al., 2011; Mazzuca et al., 2025).

Empirical evidence supports the mouth-channel specificity in the representation of abstract concepts. For example, Borghi and Zarcone (2016) presented participants with descriptive sentences referring to a concept (e.g., “Its verse is “cackle,” it has two legs and is located in farms.”) and asked them to judge whether the sentence matched a target word (e.g., “hen”), with target concepts including both concrete (e.g., “hat”) and abstract (e.g., “justice”) words. Responses were made either by pressing a key with the hand or by biting a response device with the mouth. The results revealed a significant interaction between response modality and concept type: when responding with the hand, reaction times were faster for concrete than for abstract words, whereas when responding with the mouth, no significant difference was observed between the two. In a concept-learning paradigm, participants learned novel concrete concepts (represented by Lego-based shapes) and abstract concepts (defined by spatial relationships among those shapes), and then performed a matching task; responses to abstract concepts were faster when made using the mouth than when using the hand (Granito et al., 2015). Taken together, these findings provide converging support for the WAT theory, suggesting that abstract concepts exhibit a relative advantage in the mouth-related motor channel.

According to this framework, modality-switch costs should be minimal or absent when switching between abstract verb phrases. However, the experimental results show a more nuanced pattern. In Experiment 2 (concrete verbs + abstract nouns), a general switching cost was observed, mainly in the eye channel, with Bayesian results largely inconclusive or providing moderate evidence against channel-specific effects for hand and mouth channels. In Experiment 3 (abstract verbs + abstract nouns), a significant Channel × Switch interaction was observed for the mouth channel, with moderate Bayesian evidence supporting a mouth-specific switch cost, whereas the eye channel showed evidence against such an effect and the hand channel was inconclusive.

These findings suggest that while abstract actions may preferentially engage the mouth channel, they also recruit other motor channels to some extent. The presence of switching costs for mouth-channel abstract verbs indicates that motor channel engagement is not all-or-none. Overall, the consistent observation of channel-specific switching costs across both concrete and abstract conditions implies that sensorimotor simulation is not confined to a single dominant channel. This pattern is therefore more directly consistent with the PSS framework, while WAT may better describe the preferential (Borghi et al., 2011, 2019; Borghi and Binkofski, 2014; Mazzuca et al., 2025)—but not exclusive—engagement of the mouth channel in abstract action comprehension.

### 5.2 Effects of contextual concreteness on verb embodiment

Although switch costs appeared for both concrete and abstract verbs, the embodied cognitive processes for these two types of concepts remain distinct. A comparison across the three experiments suggests that the semantic abstractness of a phrase modulates how switch costs manifest in different channels.

For concrete verbs associated with the hand channel, when paired with abstract nouns (Experiment 2), the modality-switch cost disappeared, consistent with previous findings (Schaller et al., 2017). Schaller et al. (2017) found that action videos facilitated or interfered with comprehension of sentences containing concrete verbs with concrete nouns but had little effect when concrete verbs were paired with abstract nouns, which they interpreted as a weakening of perceptual-motor simulation when concrete verbs are abstracted. In the present study, the disappearance of switch costs for these phrases may reflect a broader range of perceptual-motor simulations elicited by abstract nouns. While the hand channel was still engaged, the semantic vagueness of the abstract nouns reduced channel-specific demand, attenuating the observed modality-switch cost. Similar evidence from studies on abstract concepts such as time (Hao and Fan, 2019) and morality (Dong et al., 2018; Lu, 2021) support this view that even abstract language is embodied in perceptual and motor experience.

In the present study, the disappearance of switch costs for concrete verbs paired with abstract nouns may result from the broader range of perceptual-motor simulations elicited by abstract concepts. While these phrases still activated the hand channel, the semantic vagueness of the abstract nouns led to less channel-specific demand, reducing the observed modality-switch cost. Other research also shows that abstract action sentences involving hand movements activate motor networks associated with the hand, but processing these concepts is more complex and temporally extended (Pambuccian, 2021).

For concrete verbs associated with the mouth channel presented in concrete contexts (Experiment 1), no switch cost was observed. This may be related to the complexity of the action scenarios depicted and the richness of perceptual information in such contexts. Although these phrases were not directly related to other channels, they could still activate perceptual-motor experiences in other channels; for example, everyday activities like “背诵课文/reciting a text” or “品尝甜点/tasting a dessert” often involve concurrent hand movements. According to PSS theory, language comprehension activates multiple perceptual and motor experiences, forming simulations across the entire perceptual space (Barsalou, 1999). When the context in Experiment 2 and 3 became more abstract, the accompanying perceptual information decreased, and the perceptual-motor experiences relied upon for understanding mouth-channel verbs became relatively limited and specific, resulting in the emergence of a switch cost.

Taken together, these results indicate that the degree of contextual abstractness constrains the diversity and richness of perceptual-motor experiences during action simulation, thereby affecting the channel-specificity of motor activation and producing differential modality-switch costs across the three experiments. Although the PSS framework explains these effects, it is also compatible with a nuanced interpretation of WAT, wherein abstract actions preferentially engage the mouth channel while still recruiting other motor channels to a lesser extent. Future research could further validate the robustness of channel-switching effects by employing a more diverse set of linguistic materials and incorporating physiological measures, such as electromyography (EMG), eye-tracking, or oral muscle activity.

## 6 Conclusion

(1) Concrete verbs primarily rely on perceptual-motor simulation within their corresponding action channels, and significant switch costs are observed regardless of whether they are paired with concrete or abstract nouns.
(2) The comprehension of abstract verbs also depends on perceptual-motor simulation, as evidenced by significant switch costs.
(3) The Words as Social Tools theory predicts a mouth-channel advantage for abstract verbs, while the Perceptual Symbol Systems theory accounts for the channel-specific switch costs observed across verbs.

## Data Availability

The raw data supporting the conclusions of this article will be made available by the authors, without undue reservation.
