# Supplementary material for: Action channel switching costs in the comprehension of concrete and abstract mandarin verbs
Source: Front Psychol. 2026 Apr 28;17:1796806. doi: 10.3389/fpsyg.2026.1796806 (PMC13160815; doi:10.3389/fpsyg.2026.1796806)
Supplement: Supplementary file 1 [file Table_1.DOCX]

Supplementary Material

# Phrases used in Experiment

| 实验1使用的短语 | | | | | | | | |
| --- | --- | --- | --- | --- | --- | --- | --- | --- |
| **眼部** | 围观打斗 | 仰望天空 | 围观演出 | 凝视花朵 | 巡视领地 | 遥望夜空 | 瞄准靶子 | 撞见朋友 |
|  | 观看比赛 | 瞥见答案 | 俯视山脚 | 观察蚂蚁 | 巡视仓库 | 注视茶杯 | 直视双眼 | 盯住屏幕 |
| **手部** | 签署协议 | 签署名字 | 托举婴儿 | 摇晃水杯 | 捧起清水 | 摘取苹果 | 打开房门 | 抚摸手背 |
|  | 握住长剑 | 摘掉花朵 | 握住长剑 | 挪动茶几 | 捏住薯片 | 举起牌子 | 抓住衣角 | 触摸皮肤 |
| **口部** | 辱骂顾客 | 吆喝买卖 | 噘嘴生气 | 歌唱乐曲 | 高呼口号 | 怒斥奸商 | 训斥下属 | 朗诵诗歌 |
|  | 背诵古文 | 呼喊仆人 | 吮吸乳汁 | 品尝甜点 | 咀嚼香蕉 | 亲吻脸颊 | 咽下口水 | 吞服胶囊 |

| 实验2使用的短语 | | | | | | | | |
| --- | --- | --- | --- | --- | --- | --- | --- | --- |
| **眼部** | 监视思想 | 眺望未来 | 瞄准机遇 | 俯视众生 | 仰望伟人 | 遥望故乡 | 观察本质 | 遥望未来 |
| **手部** | 播种希望 | 拥抱自然 | 推动进步 | 拉长战线 | 拨开迷雾 | 推到假设 | 抓住精髓 | 捕捉情绪 |
| **口部** | 呼唤亲情 | 咽下苦果 | 歌唱幸福 | 品尝苦果 | 吹散阴霾 | 歌唱明天 | 咽下委屈 | 吮吸养分 |

| 实验3使用的短语 | | | | | | |
| --- | --- | --- | --- | --- | --- | --- |
| **眼部** | 展望未来 | 盼望和平 | 洞察人心 | 看重结果 | 轻视对手 | 忽视缺点 |
|  | 歧视学历 | 鄙视坏人 | 回顾历史 | 漠视事实 | 藐视法制 | 关注内心 |
| **手部** | 掌握权力 | 托付终身 | 触动内心 | 把握方向 | 摸透底细 | 给与补偿 |
|  | 把持朝政 | 扶持企业 | 掌握规律 | 抛弃使命 | 摸透脾气 | 摸透心思 |
| **口部** | 品味人生 | 吃透课本 | 呼吁和平 | 鼓吹歪理 | 提倡节约 | 空谈理想 |
|  | 品味故事 | 鼓吹谬论 | 歌颂生活 | 煽动矛盾 | 提倡民权 | 诽谤名誉 |

# Phrases used in Experiment(English-translated)

| Phrases used in Experiment 1 | | | | | | | | |
| --- | --- | --- | --- | --- | --- | --- | --- | --- |
| **Eye-related phrases** | watch a fight | gaze at the sky | watch a performance | stare at flowers | survey territory | look into the night sky | aim at a target | run into a friend |
|  | watch a match | glimpse an answer | look down at the mountainside | observe ants | inspect a warehouse | fixate on a teacup | look into eyes | stare at a screen |
| **Hand-related phrases** | sign an agreement | sign a name | lift a baby | shake a cup | hold water | pick an apple | open a door | stroke a hand |
|  | grip a sword | pluck a flower | grip a sword | move a coffee table | pinch a chip | raise a sign | grab a sleeve | touch skin |
| **Mouth-related phrases** | insult a customer | shout to attract buyers | pout in anger | sing a song | chant a slogan | rebuke a merchant | scold a subordinate | recite a poem |
|  | recite classical texts | call a servant | suck milk | taste dessert | chew a banana | kiss a cheek | swallow saliva | swallow a capsule |

| Phrases used in Experiment 2 | | | | | | | | |
| --- | --- | --- | --- | --- | --- | --- | --- | --- |
| **Eye-related phrases** | monitor thoughts | look toward the future | aim at opportunities | look down on humanity | look up to great figures | gaze toward the homeland | observe essence | look into the future |
| **Hand-related phrases** | sow hope | embrace nature | promote progress | extend the battle line | clear away the fog | overturn a hypothesis | grasp the essence | capture emotions |
| **Mouth-related phrases** | call for family affection | swallow bitterness | sing happiness | taste bitterness | blow away the gloom | sing tomorrow | swallow grievance | suck nourishment |

| Phrases used in Experiment 3 | | | | | | |
| --- | --- | --- | --- | --- | --- | --- |
| **Eye-related phrases** | envision the future | hope for peace | perceive the human mind | value outcomes | despise opponents | ignore flaws |
|  | discriminate against education | despise evildoers | review history | disregard facts | contempt the rule of law | attend to the inner self |
| **Hand-related phrases** | grasp power | entrust a lifetime | touch the inner self | grasp direction | probe the truth | grant compensation |
|  | control state affairs | support enterprises | grasp principles | abandon a mission | probe temperament | probe intentions |
| **Mouth-related phrases** | savor life | digest textbooks | appeal for peace | promote fallacies | advocate frugality | talk empty ideals |
|  | savor stories | promote false doctrines | praise life | incite conflict | advocate civil rights | slander reputation |
